# Investigating Host Preference of Root Endophytes of Three European Tree Species, with a Focus on Members of the *Phialocephala fortinii*—*Acephala applanata* Species Complex (PAC)

**DOI:** 10.3390/jof7040317

**Published:** 2021-04-19

**Authors:** Sophie Stroheker, Vivanne Dubach, Irina Vögtli, Thomas N. Sieber

**Affiliations:** 1 Swiss Federal Institute for Forest, Snow and Landscape Research WSL, Swiss Forest Protection, Zürcherstrasse 111, 8903 Birmensdorf, Switzerland; vivanne.dubach@wsl.ch (V.D.); irina.voegtli@wsl.ch (I.V.); 2 ETH Zurich, Institute of Integrative Biology, Forest Pathology and Dendrology, Universitätstrasse 16, 8092 Zurich, Switzerland; thomas.sieber@env.ethz.ch

**Keywords:** *Picea abies*, *Fraxinus excelsior*, *Acer pseudoplatanus*, *Ilyonectria*, *Neonectria*, *Mycosphaerellaceae*, microsatellite genotyping

## Abstract

Host preference of root endophytes of the three European tree species of Norway spruce (*Picea abies*), common ash (*Fraxinus excelsior*), and sycamore maple (*Acer pseudoplatanus*) were investigated in two forest stands near Zurich, Switzerland. The focus was placed on members of the *Phialocephala fortinii* s.l. (sensu lato)—*Acephala applanata* species complex (PAC), as well as other dark septate endopyhtes (DSE). PAC species were identified based on 13 microsatellite loci. Eleven PAC species were found, with *Phialocephala helvetica*, *P. europaea* being the most frequent. All but cryptic species 12 (CSP12) preferred Norway spruce as a host. Though very rare in general, CSP12 was most frequently isolated from maple roots. Regarding the abundant PAC species, *P. helvetica* and *P. europaea*, the preference of spruce as a host was least pronounced in *P. europaea*, as it was also often isolated from ash and maple. It is the first record of PAC found on common ash (*Fraxinus excelsior*). *Cadophora orchidicola*, a close relative of PAC, has frequently been isolated from ash. Various species of the *Nectriaceae* (*Cylindrocarpon* spp.) have often been isolated, particularly from maple roots. By comparison, *Pezicula* spp. (*Cryptosporiopsis* spp.) was found to be abundant on all three hosts. *Phomopsis phaseoli* exhibits a clear preference for spruce.

## 1. Introduction

Mycorrhizal fungi tend to be associated with roots of certain tree species. Many ectomycorrhizal fungi are associated with multiple tree species [1,2], whereas representatives of the genera *Suillus* and *Rhizopogon* are *Pinaceae*-specific [3]. In cases when a fungal taxon occurs exclusively on one host, the term “host-specificity” is used [4]. The relationships between fungi and host are also known to be less specific. In such cases, “host preference” is more appropriate, meaning that the fungus prefers one host over another [5]. For mycorrhizal fungi, strong host preference was apparent for many species [6,7], but the contrary is also true for many other species [8,9].

In addition to mycorrhizal fungi, woody roots are colonized by a plethora of endophytic fungi. In contrast to mycorrhizal fungi, which colonize only primary roots, endophytic fungi occur throughout the entire root system. Nevertheless, host specificity and host preference of endophytic fungi have not been as well studied as those of mycorrhizal fungi. Dark septate endophytes (DSE), i.e., species that form dark, septated mycelia in culture, and Non-DSE, i.e., species without darkly pigmented hyphae, manifest as root endophytes [10,11,12,13]. The DSE are mainly *Cadophora* species and representatives of the the *Phialocephala fortinii* s.l. (sensu lato)—*Acepahala applanata* species complex (PAC) [14]. The Non-DSE mostly belong to the genera *Gibberella* (*Fusarium*), *Ilyonectria*, *Neonectria* (*Cylindrocapron*) species, and some are representatives of the *Sebacinales*. In addition, *Pezicula* (*Cryptosporiopsis*) species are common and are classified as DSE or Non-DSE depending on the species and the age of the mycelium. Depending on the plant family, either DSE or Non-DSE dominate. According to current knowledge, DSE tend to dominate on *Pinaceae,* while, on deciduous woods (numerous families) Non-DSE dominate (see Figure 38.1 in Sieber and Grünig [15]).

DSE have been identified in the roots of over 600 different plant species [12], ranging from subtropical to arctic environments [10,16,17,18,19]. Their broad host range and substantial abundance suggest that these fungi play an important, though as yet largely unknown, role in various ecosystems [12,20]. For example, it has been demonstrated that DSE have the potential to facilitate nutrient absorption in their host plants, as well as increase their resistance to toxic environmental conditions [21,22,23,24].

Probably the best-known endophytes present in woody roots are the representatives of the PAC, which have mainly been colonizing the roots of conifers and ericaceous plants all across the Northern hemisphere [13,25,26,27,28,29]. So far, 21 morphologically indistinguishable cryptic species (CSP) have been identified, of which eight have been formally described: *Phialocephala turicensis* (CSP1), *P. letzii* (CSP2), *P. europaea* (CSP3), *P. helvetica* (CSP4), *P. uotilensis* (CSP5), *P. subalpina* (CSP6), *P. fortinii* s.s., and *Acephala applanata* [14,30,31,32,33]. There is evidence of the discovery of a 22nd CSP found in the Pfynwald—a forest in Switzerland dominated by Scots pine (*Pinus sylvestris*) [34]. PAC communities are highly diverse and the factors governing community assembly remain unknown [28,35,36,37], as do their modes of reproduction and dispersal. Despite evidence for sexual reproduction, a sexual state has never been witnessed [16,38], leaving long distance transfer with colonized plant material as one possible explanation for local diversity [39,40].

Very few studies on DSE involve non-coniferous plants, but DSE (including PAC) have also been detected in the roots of deciduous trees such as beech (*Fagus sylvatica*), sessile oak (*Quercus petraea*), and common oak (*Quercus robur*) [11,41]. However, the dominant DSE in deciduous trees appear to be *Pezicula* and *Cadophora* species rather than PAC. In oak roots (*Q. robur* and *Q. petraea*), *Pezicula melanigena*, *P. radicicola* and *Cadophora fastigiata* dominate the root endophyte community [41]. Likewise, *P. radiciola* was the most frequently presenting endophyte in roots of European beech (*Fagus sylvatica*) [11]. Accordingly, data pertaining to Non-DSE in deciduous trees are also scarce. In oak roots (*Q. petraea* and *Q. robur*) only *Ilyonectria radicicola* and one species of *Cystodendron* were pervasive [41]. The Non-DSE *Cylindrocarpon didymum* was the second most present endophyte in *F. sylvatica* roots [11]. Zheng [42] reported the detection of a *Neonectria* species in roots of Ginkgo (*Gingko biloba*). Even if *G. biloba* resembles a deciduous tree in its habitus, it is classified as a gymnosperm. Phylogenetically, *G. biloba* is positioned between conifers and deciduous trees, and one would expect it to be colonized by both conifer-specific and deciduous-tree-specific endophytes. However, this requires more extensive research. Non-DSE, i.e., *Fusarium* and *Neonectria* species, have also been detected in roots of wild cherry (*Prunus avium*) [43].

In the present study, we set out to investigate the degree to which roots of deciduous tree species growing in forest stands with a high rooting density of Norway spruce (*Picea abies*) are colonized by PAC, Non-PAC DSE and Non-DSE fungi. Mixed forest stands with Norway spruce-dominated canopies and understories dominated by deciduous tree species, in particular common ash (*Fraxinus excelsior*) and sycamore maple (*Acer pseudoplatanus*) were chosen as locations for the study.

## 2. Materials and Methods

### 2.1. Study Site

Two Norway spruce (*Picea abies* L. Karst) forest stands were selected in the vicinity of Zurich, Switzerland (Site 1: N 47°22′55′′, E 8°28′1.9′′, 532 m MSL.; Site 2: N 47°23′31.9′′ E 8°33′55′′, 628 m MSL), both showing dense undergrowth of common ash (*Fraxinus excelsior* L.) and sycamore maple (*Acer pseudoplatanus* L.).

### 2.2. Sampling Design

A sampling grid consisting of nine grid points (6 m × 6 m; distance of 3 m between grid points) was established at each study site. Sampling of Norway spruce, common ash and sycamore maple took place within a 1 m radius of each grid point. Within each circle, 5 small ash trees and 5 small maple trees (~20 cm in height) were carefully excavated. Additionally, root complexes of mature Norway spruce trees were collected at 5 sampling points within each circle. All plant material was placed in plastic bags and stored at 4 °C in the refrigerator until further processing within the following 2–3 days.

### 2.3. Isolation of Fungal Cultures

The root systems of the collected ash and maple, as well as the spruce root pieces, were carefully washed under running tap water to remove all soil particles. Afterwards, 10 root segments (approx. 5 cm in length) with varying diameters (approx. 1–5 mm) were removed per tree or root complex (spruce), respectively, and surfaces were sterilized in accordance with Ahlich and Sieber [11]. In brief: immersion of root segments in 99% (*v*/*v*) ethanol for 1 min, followed by 5 min in 35% (*v*/*v*) hydrogen peroxide and, finally, 30 s in 99% (*v*/*v*) ethanol. A smaller root segment of 0.5 cm was excised aseptically from the midpoint of each surface sterilized root, placed on terramycin malt agar (TMA; 20 g/L malt extract, 16 g/L agar, 50 mg/L terramycin (active ingredient: oxytetracycline, Pfizer Ltd., Hyderabad, Telangana, India)), and incubated at 20 °C in the dark for approximately 2 weeks [11].

### 2.4. Classification of Fungal Cultures

All emerging fungal cultures were first visually divided into DSE and Non-DSE fungi based on the presence or absence of dark, melanized mycelium. DSE fungi were further divided into PAC and Non-PAC isolates based on microsatellite genotyping (described below). All the Non-DSEs were visually grouped into 4 main categories based on culture morphology: *Cylindrocarpon* spp., *Cryptosporiopsis* spp., *Phomopsis* spp., and others. The classification of *Pezicula* (*Cryptosporiopsis*) species as either DSE or Non-DSE poses a problem. Many species initially grow in culture with a colorless white mycelium. In some species, however, the colonies become darker with age. Since the assignment of the cultures to morphotypes occurred when the colonies were still relatively young, the *Pezicula* species were considered to be Non-DSE.

### 2.5. Microsatellite Genotyping of DSE Isolates

In order to clearly identify members of the PAC within the large number of DSE isolates, single-hyphal tip (SHT) cultures were prepared from all DSE emerging from the root segments and incubated in the dark for a further 2 weeks. Afterwards, DNA was extracted from each culture using the NucleoSpin^®^ 96 Plant II kit by Macherey-Nagel (Macherey-Nagel, Düren, Germany). Multiplex PCR and fragment analysis of 14 microsatellite loci relevant for CSP assignment was performed, as described in Queloz et al. [44].

The obtained microsatellite data were analyzed using the software GeneMapper^®^ (v. 4.0, Applied Biosystems, Waltham, MA, USA). Samples showing two peaks at any of the loci were considered diploid, and therefore removed from the dataset. The software GeneClass2 (v. 2.0, INRA/CIRAD 2003, Paris, France, [45]) was used for assignment tests for CSP recognition. Since GeneClass2 only uses a subset of allele data for species assignment, all samples classified with a probability below 95% were manually compared to our PAC database containing approximately 5000 records.

### 2.6. Sequencing of Non-PAC DSE and Non-DSE Cultures

The internal transcribed spacer (ITS) region of the nuclear rDNA of all Non-PAC DSE and a selection of representative isolates of Non-DSE fungi were sequenced [46]. A detailed protocol of all steps can be found in [47]. The sequences were BLASTed against GenBank.

### 2.7. Statistical Analyses

Differences between the three tree species with respect to endophytic root colonization were tested pairwise for statistical significance using the Wilcoxon–Mann–Whitney test [48,49]. All statistical tests were performed using the software R Version 3.1.3 (R Development Core Team, Vienna, Austria, 2017).

## 3. Results

A total of 1350 root segments were obtained at each sampling site, resulting in a total of 2700 root segments being plated for fungal growth. Of these 2700 root segments, 651 (24%) showed no fungal growth and were considered sterile. Amongst the remaining 2049 (76%) segments, DSE grew from 615 segments. With the remaining 1434 segments, we observed Non-DSE fungi emerge (Table 1).

### 3.1. PAC and Non-PAC DSE

#### 3.1.1. PAC

The PAC-specific microsatellites were successfully amplified in 501 of the 615 DSE isolates (Table 2). PAC was most frequently isolated from spruce (426; 47.3%). Only few isolates were obtained from maple (38; 4.2%) and ash (37; 4.1%). The PAC isolates belonged to eleven different cryptic species (CSP). Clearly, *Phialocephala helvetica* was found to be the most abundant CSP, followed by *Phialocephala europaea* (Table 2). Five CSPs (*Phialocephala subalpina, Phialocephala fortinii* s.s. (senso stricto), CSP8, CSP14, and *Acephala applanata*) were only found once, and, thus, only on one particular tree species. CSP13 was found twice on spruce. An additional 32 isolates were also PAC, but not assignable to any CSP because too few loci amplified successfully, and eight isolates were diploid. For 74 DSE isolates, the microsatellite method did not work at all, and, therefore, they were considered Non-PAC DSE.

PAC colonization rate was 1.5 times higher at site one (22% of the root segments colonized) than at site two (15%). *Phialocephala turicensis, Phialocephala letzii, P. europaea*, and *P. helvetica* were found at both sites and on all three tree species. CSP12 was isolated from all tree species, but solely at site one. *P. subalpina* and CSP 14 were only found at site one and solely on maple. *P. fortinii* s.s., and CSP13 only occurred on spruce at site one. CSP8 also occurred only on spruce at site two. *A. applanata* was only found on ash and only at site one. A total of 10 CSPs were found at site one, whereas only 5 were found at site two (Table 2).

**Figure 1:**
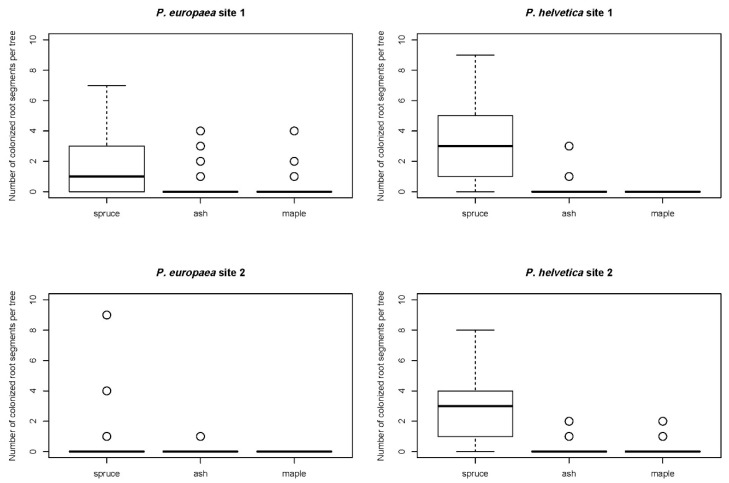
Number of root segments colonized by *Phialocephala europaea* (**left**) and *Phialocephala helvetica* (**right**) per tree for spruce, ash, and maple at sites 1 and 2 (*n* = 10 per tree).

The number of colonized root segments per tree was investigated for the two most abundant CSPs, *P. europaea* and *P. helvetica* (Figure 1). Regarding both *P. europaea* and *P. helvetica*, spruce colonization was significantly higher than that of maple and ash, whereas maple and ash were in line with each other (Figure 1, Table 2, Table 3 and Table 4).

#### 3.1.2. Non-PAC DSE

Sequencing of the ITS regions was successful for 64 (of 74), DSEs for which microsatellite analysis did not work at all and which were thus considered Non-PAC DSE. Of these 64 DSE, only one (*Phoma* sp.) was from spruce, 49 from ash and 14 from maple. The similarity of the 10 isolate sequences with existing ones deposited in GenBank was below 93 % and, as a result, these isolates could not be identified. The similarity of the remaining 54 isolates exceeded 98 %, with at least one sequence found in GenBank, and could at least be identified at the genus level. The most frequent Non-PAC DSE was a species of *Phoma*, an anamorphic form of a *Mycosphaerellaceae*, followed by *Cadophora orchidicola* (Table 5, Table S1).

*C. orchidicola* and *Phoma* sp. were more frequently isolated from ash than from maple. At site one, *C. orchidicola* could not be detected on maple, solely on ash (Table 5). Another species of *Cadophora* was rarely observed on ash and maple and two isolates of a *Rhexocercosporidium* species occurred on ash (Table S1).

### 3.2. Non-DSE

Almost half of the Non-DSE isolates belonged to one of the following three taxa: *Cylindrocarpon* spp. (461 isolates), *Cryptosporiopsis* spp. (149 isolates), and *Phomopsis* spp. (67 isolates) (Table 1). The culture morphology of the other half (767 isolates) was highly diverse and no larger group of isolates with identical culture morphology could be recognized. This suggests that these isolates belong to many different species. However, since the main aim of the study was to focus on the most abundant endopyhtes, no further attempt was made to identify all these species, and they were assigned to the taxon “Others” (Table 1). Colonization by *Cylindrocarpon* spp. was significantly higher on maple than on ash or spruce (*p* < 0.0001), and that of spruce was slightly lower, yet still of significance, than that of ash (*p* = 0.049). *Cryptosporiopsis* spp. occurred equally frequently on both spruce and ash (*p* = 0.924), whereas the colonization rate of maple was lower (*p* = 0.041). Interestingly, the colonization of ash was distinctly higher at site one than at site two, whereas the opposite was true for the colonization of spruce (Table 1). *Phomopsis* spp. occurred almost exclusively on spruce at both sites (*p* < 0.0001) (Table 1).

With a few exceptions, the ITS sequences of the isolates selected from each of the three most abundant morphotypes, *Cylindrocarpon* spp., *Cryptosporiopsis* spp. and *Phomopsis* spp., showed that the isolates had been assigned to the correct morphotype based on culture morphology. Isolates of morphotype “*Cylindrocarpon* spp.” belonged to at least four different species: *Cylindrocarpon* sp., *Ilyonectria destructans*, *Ilyonectria* sp. and *Neonectria* sp. (Table S1). Isolates of morphotype “*Cryptosporiopsis* spp.” belonged to at least three different species: *Pezicula ericae*, *P. melanigena* and *P. radicicola*. In contrast, all the isolates selected for sequencing from *Phomopsis* spp. belonged to the same species: *Phomopsis phaseoli* (Table S1).

## 4. Discussion

### 4.1. PAC and Non-PAC DSE

#### 4.1.1. PAC

Our results indicate a clear preference of PAC for Norway spruce as a host. Ash and maple were only very scarcely colonized. As far as we know, this is the first report of PAC in roots of common ash. Despite intensive sampling, Menkis [50] never found *Phialocephala* spp. in *Fraxinus excelsior*. Possibly, maple and ash are only colonized by PAC due to the density of Norway-spruce roots heavily colonized by PAC being very high, making the transfer of PAC through root contact highly probable. It has been experimentally shown that PAC is transferred preferentially via root contact [51]. Ash and maple roots from conifer-free stands must be studied in more detail to prove this hypothesis.

Looking at the individual PAC species, it is noticeable that *P. helvetica* has a clear preference for Norway spruce. In contrast, *P. europaea* is only the second most frequent PAC species in spruce roots, but the most frequent PAC species in the roots of the two deciduous hosts, though at a much lower frequency. Thus, *P. europaea* is certainly the least host-specific PAC species in this study. A very similar picture was found in another study comparing PAC in pine (*Pinus sylvestris*) and oak (*Quercus pubescens*) roots. The dominant species was again *P. Helvetica*, with a distinct preference for *Pinus sylvestris*. The second most common species was *P. europaea*, which was more common on pine, but the difference between pine and oak root colonization was far less pronounced than that for *P. helvetica* [34]. Halmschlager and Kowalski [41] have demonstrated that PAC (*P. fortinii* s.l.) were quite rare in oak roots (*Quercus robur* and *Q. petraea*). In their study, the majority of endopyhtes in oak roots were species of *Cadophora*, *Cylindrocarpon* (*Ilyonectria*, *Neonectria*), *Cryptosporiopsis* (*Pezicula*) and *Cystodendron* (*Mollisia*) [41], which is similar to the observations made within the scope of this study for ash and maple.

*Phialocephala subalpina* is considered non-host-specific since it has been isolated from 16 different plant species thus far [15]. *P. subalpina* is likely the most common PAC species in the world. Interestingly, it was found only once on maple in this study, and also very rarely in the study of Landolt et al. [34]. Some patterns of host preference also emerged from three undisturbed, subalpine forest sites in Switzerland, where the roots of *Vaccinium* spp. and *Picea abies* had been examined [38,52]. *Acephala applanata* was almost exclusively isolated from *P. abies*, whereas *P. subalpina* showed a preference for *Vaccinium* spp. but also occurred frequently on *P. abies*. Again, host preference of *P. europaea* was less pronounced. Over 70% of the *P. europaea* isolates originated from *P. abies* located at one of the three sites, whereas the repartition between *Vaccinium* spp. and *P. abies* was about 50:50 at the other two sites [38,52]. The reasons for this disparity in repartition can be attributed to climatic and edaphic differences or on-habitat occupation by competitors.

Evaluation of host preference/specificity of PAC species based on field observations is potentially hampered by confounding factors such as environmental parameters (climatic and edaphic conditions) and the presence–absence of competitors, including other PAC species. Thus, one can only speculate as to the relative effects of host, environment, and competitors on PAC host preference. Moreover, PAC do not spread through the air. Thus, if a PAC species is not present, potential hosts remain clean of the species. For example, *P. subalpina* is very rare in the region of Zurich, where many of our studies were conducted [35,37]. Consequently, it is absent or very rare regardless of the presence of the host. The name “subalpina” was assigned to this species because it was mainly found in subalpine regions of Switzerland [14]. However, more recent results show that the species is also highly abundant at lower elevations (600–700 m a.s.l.), e.g., in the Allgäu, Germany [40]. Thus, the absence of *P. subalpina* in the region of Zurich is presumably not due to low altitude, but, rather, that both sites were deforested at the end of the 19th century and only reafforested thereafter, probably with trees whose roots had not been colonized by *P. subalpina*.

The relative influence of host and environmental factors (climatic and edaphic conditions) on ectomycorrhizal (ECM) communities has been widely discussed. Kennedy et al. [2] found 39 out of a total of 56 ectomycorrhizal fungi to be host-specific on either *Pseudotsuga menziesii* or *Lithocarpus densiflora*. Similarly, the combination of molecular identification of plants and ectomycorrhizal fungi in the Yasuni National Park (Ecuador) revealed a significant degree of host preference within the most common fungal species [53]. In contrast, only 8 of the 205 ectomycorrhizal species proved to be strictly host-specific in one of eight tree species in a study conducted by Ishida et al. [6]. Dickie [54] concluded that ECM are not definitively host-specific since there are no physiological or anatomical obstacles. The question as to whether host preference reflects, rather, the influence of environmental factors, can only be answered by in vitro experiments under controlled environmental conditions.

A comparison of data gathered for PAC with those available for ECM would be worthwhile, as PAC have been reported as co-inhabitants of ECM [27] (Summerbell, 2005), or even forming ECM themselves [55], as does *Acephala macrosclerotiorum*, a close relative of PAC, on *P. sylvestris* [56]. However, in contrast to mycorrhizal fungi, PAC do not only colonize primary roots but can be found throughout the root system.

#### 4.1.2. Non-PAC DSE

With the exception of two *Rhexocercosporidium* species on ash, all Non-PAC DSE were either *Phoma* sp. or *Cadophora orchidicola*. Together with PAC and *C. orchidicola*, *Phoma* sp. was one of the most common endophytes found in the roots of ash and maple in this study. It would certainly be interesting to learn more about the biology of this species. In our study, *C. orchidicola* displayed a preference for ash and was not present on spruce. The scarcity of PAC in ash roots could be partly due to the fungicidal activity of *C. orchidicola* (see below). Conflicting this hypothesis is the fact that PAC is also rarely detected on sycamore maple, although *C. orchidicola* was found only once in maple roots.

*Cadophora orchidicola* was originally described as a root endophyte of the orchid *Platanthera hyperborean* [57]. However, the fungus has also been found as an endophyte in the roots of various other plants from various plant families, such as *Littorella uniflora* (Plantaginaceae), a plant found on the sandy shores of freshwater lakes [58], *Kalimeris indica* (Asteraceae) [59], *Colobanthus quitensis* (Caryophyllaceae), and *Deschampsia antarctica* (Poaceae) in Antarctica [60]. The effects of *C. orchidicola* on their hosts are not fully understood. While the roots of *Salix glauca* (Salicaceae) became significantly longer through inoculation with *C. orchidicola* [61], no comparable effect was observed for roots of *Saussurea involucrata* (Asteraceae) [62]. However, *S. involucrata* inoculated with the fungus grew to be significantly larger and had a greater biomass. The metabolite cercosporamide, produced by *C. orchidicola*, was shown to possess fungicidal properties against *Pestalotia diospyri*, *Botrytis cinerea, Fusarium oxysporum, Sclerotium rolfsii*, and *Penicillium digitatum* [59].

### 4.2. Non-DSE

The taxon *Cylindrocarpon* spp. included at least three different species: *Ilyonectria destructans* (anamorphic form: *Cylindrocarpon destructans*), *Ilyonectria* sp., and *Neonectria* sp. (see Table S1 for sequence information). *I. destructans* is often more common in the roots of deciduous trees than conifer roots [11]. This finding is also true for sycamore maple and spruce in the present study. However, if we include ash, we see that the infestation level of ash is about the same as that of spruce (Table 1). This means that, within the parameters of this study, *I. destructans* is generally not more common in roots of deciduous trees than conifers. According to the literature, *I. destructans* is often found on and in the roots of a large number of plant species, and is also prevalent in soil [63]. *I. destructans* is usually associated with root dieback, but information on the virulence of this species in the existing literature is contradictory. Pathogenicity tests were often carried out on young seedlings [64,65], and virulence depended strongly on the isolate [66,67]. Similarly, isolates of *I. destructans* demonstrated a high incidence of sequence divergence, suggesting the existence of several phylogenetic species in this complex [68]. *I. destructans* was one of the most frequent endophytes in roots of *Quercus petraea* and *Q. robur* but it occurred 1.5 times more frequently in the living roots of declining trees than in those of healthy trees [41]. Consequently, Halmschlager and Kowalski [41] considered *I. destructans* a weak pathogen. Interestingly, the occurrence of *I. destructans* was inversely proportional to that of PAC (*Phialocephala fortinii* s.l.), possibly because of antagonistic behaviour between the two.

*Cryptosporiopsis* spp. are anamorphic forms of the *Pezicula* species. We found the following three species: *Pezicula ericae, P. melanigena* and *P. radiciola*. *P. melanigena* was the most common. As far as we know, *P. radiciola* and *P. melanigena* have, so far, only been witnessed in oak roots [41,69,70]. Spruce, ash, and sycamore maple would therefore be three newly discovered hosts for these two species. The same may be true for *P. ericae*, which has been uncovered in the roots of various ericaceous plant species [71]. We have identified these species, once each on ash and sycamore maple (Table S1). All three species grow initially as white colorless mycelia which, however, become dark with age. Thus, these three species could also be considered as DSE. However, it would be interesting to know whether older endophytic mycelium of these species also appears dark in the roots. For PAC, we know that the endophytic mycelium is always melanized [72].

*Phomopsis phaseoli* was almost exclusively found in spruce roots, where it likely supports PAC in the control of root pathogens [73]. *P. phaseoli* (*Diaporthe phaseolorum*) is actually known to be a causal agent of pod and stem blight of soybean. With one exception, the ITS sequences of our *P. phaseoli* isolates are, however, in sound agreement (>98.2% similarity) with those of an isolate from leaves of an *Espeletia* (*Asteraceae*) collected at 3250 m above sea level in the Colombian Andes [74]. These *P. phaseoli* isolates from *Espeletia* spp. were shown to strongly inhibit growth of *Phytophthora infestans*, the late blight pathogen affecting potato plants, in dual cultures [74].

It will be exciting to study the host preference of PAC in other forest communities. The choice of sites would also have to take into account conifer-free deciduous forests.

## Figures and Tables

**Table 1:** The number and the percentage of isolates of the morphotypes Dark septate endophytes (DSE), *Cylindrocarpon*, *Cryptosporiopsis*, *Phomopsis*, others, and the number and percentage of sterile root segments (no fungal growth) for each host tree species at each site. The percentages refer to the 450 root segments (=100%) examined per host and site.

	Host	Sterile		DSE ^1^		*Cylindrocarpon* spp.		*Cryptosporiopsis* spp.		*Phomopsis* spp.		Others	
		n	%	n	%	n	%	n	%	n	%	n	%
**Site 1**	Spruce	49	10.9	268	59.6	43	9.6	12	2.7	42	9.3	38	8.4
	Ash	164	36.4	63	14.0	44	9.8	44	9.8	4	0.9	131	29.1
	Maple	82	18.2	51	11.3	151	33.6	13	2.9	1	0.2	156	34.7
	Total	295	21.9	382	28.3	238	17.6	69	5.1	47	3.5	325	24.1
**Site 2**	Spruce	58	12.9	210	46.7	41	9.1	47	10.4	20	4.4	78	17.3
	Ash	201	44.7	19	4.2	66	14.7	16	3.6	0	0.0	155	34.4
	Maple	97	21.6	4	0.9	116	25.8	27	6.0	0	0.0	209	46.4
	Total	356	26.4	233	17.3	223	16.5	90	6.7	20	1.5	442	32.7
Total	all	651	24.1	615	22.8	461	17.1	159	5.9	67	2.5	767	28.4
TOTAL: 2720 ^2^ (100.7%)													

^1^ Dark septate endophytes including PAC; ^2^ Total 2720 > 2700 root segments: few occasions with more than one isolate growing from a single root segment.

**Table 2:** The number and the percentage of isolates per PAC species isolated from spruce, maple, and ash, as well as the total number and the percentage of isolates for both sites. Further, the sum of isolates and species for each tree species. (*n* = 450 root segments per host and site).

	Spruce						Ash						Maple						Totals	
PAC ^1^ Species	Site 1		Site 2		Total		Site 1		Site 2		Total		Site 1		Site 2		Total		Total Isolates	
	n	%	n	%	n	%	n	%	n	%	n	%	n	%	n	%	n	%	n	%
*P. turicensis* (CSP11)	9	2	6	1.3	15	1.7	0	0	1	0.2	1	0.1	2	0.4	0	0	2	0.2	18	0.67
*P. letzii* (CSP2)	3	0.7	13	2.9	16	1.8	0	0	1	0.2	1	0.1	1	0.2	0	0	1	0.1	18	0.67
*P. europaea* (CSP3)	80	17.8	21	4.7	101	11.2	18	4	1	0.2	19	2.1	21	4.7	0	0	21	2.3	141	5.22
*P. helvetica* (CSP4)	146	32.4	143	31.8	289	32.1	6	1.3	8	1.8	14	1.6	0	0	3	0.7	3	0.3	306	11.33
*P. subalpina* (CSP6)	0	0	0	0	0	0	0	0	0	0	0	0	1	0.2	0	0	1	0.1	1	0.04
*P. fortinii* s.s. (CSP7)	1	0.2	0	0	1	0.1	0	0	0	0	0	0	0	0	0	0	0	0	1	0.04
CSP8	0	0	1	0.2	1	0.2	0	0	0	0	0	0	0	0	0	0	0	0	1	0.04
CSP12	1	0.2	0	0	1	0.1	1	0.2	0	0	1	0.1	9	2	0	0	9	1	11	0.41
CSP13	2	0.4	0	0	2	0.2	0	0	0	0	0	0	0	0	0	0	0	0	2	0.07
CSP14	0	0	0	0	0	0	0	0	0	0	0	0	1	0.2	0	0	1	0.1	1	0.04
*A. applanata*	0	0	0	0	0	0	1	0.2	0	0	1	0.1	0	0	0	0	0	0	1	0.04
Sum isolates	242	53.8	184	40.9	426	47.3	26	5.8	11	2.4	37	4.1	35	7.8	3	0.7	38	4.2	501	18.56
Sum species	7		5		8		4		4		6		6		1		7		11	

^1^ Phialocephala fortinii s.l. (sensu lato)—Acephala applanata species complex.

**Table 3:** Wilcoxon–Mann–Whitney test results (*p*-values) for the pairwise comparisons of the three hosts in regard to the frequency of colonization by *Phialocephala europaea*. The values for site 1 are in the upper-right half of the table, those for site 2 are in the lower-left half of the table.

	Spruce	Ash	Maple
**Spruce**	.	0.0008 **	0.002 *
**Ash**	0.0039 *	.	0.7476
**Maple**	0.0009 **	0.3282	.

Signif. codes: ** 0.001 < *p* ≤ 0.01; * 0.01 < *p* ≤ 0.05; 0.05 < *p* ≤ 0.1.

**Table 5:** DSE colonization of ash and maple at site 1 and site 2. Data on PAC colonization see Table 2. Data for spruce not shown.

Taxon	Site 1		Site 2	
	Ash	Maple	Ash	Maple
**PAC**	26	35	11	3
***C. orchidicola***	13	-	6	1
***Phoma*** **sp.**	27	6	1	7
***Rhexocercosporidium*** **sp.**	2	-	-	-

**Table 4:** Wilcoxon–Mann–Whitney test results (*p*-values) for the pairwise comparisons of the three hosts in regard to the frequency of colonization by *Phialocephala helvetica*. The values for site 1 are in the upper-right half of the table, those for site 2 are in the lower-left half of the table.

	Spruce	Ash	Maple
**Spruce**	.	<0.0001 ***	<0.0001 ***
**Ash**	<0.0001 ***	.	0.1596
**Maple**	<0.0001 ***	0.1478	.

Signif. codes: *** *p* ≤ 0.001; 0.05 < *p* ≤ 0.1.

## Supplementary Materials

The following are available online at https://www.mdpi.com/article/10.3390/jof7040317/s1, Table S1: Strains of which the DNA ITS region was sequenced, with closest matching sequence in GenBank and GenBank accession numbers of the new sequence.
