# Supplementary material for: Investigating Host Preference of Root Endophytes of Three European Tree Species, with a Focus on Members of the Phialocephala fortinii—Acephala applanata Species Complex (PAC)
Source: J Fungi (Basel). 2021 Apr 19;7(4):317. doi: 10.3390/jof7040317 (PMC8073920; doi:10.3390/jof7040317)
Supplement: Supplementary file 1 [file jof-07-00317-s001.zip › jof-1168903-supplementary.pdf]

Journal of *Fungi*

Supplementary Materials: Investigating Host Preference of Root Endophytes of Three European Tree Species, with a Focus on Members of the *Phialocephala fortinii—Acephala applanata* Species Complex (PAC)

Table S1. Strains of which the DNA ITS region was sequenced, with closest matching sequence in GenBank and GenBank accession numbers of the new sequence.

Strain	Host	Species[1]	GenBank Accession Number	Minimum Identity	Maximum Identity	Best Match in GenBank
P16Es2_1	*Fraxinus excelsior*	*Agrocybe erebia*	MT107606	96	99.8	MK573902
P7Ah1_6	*Acer pseudoplatanus*	*Cadophora orchidicola*	MT107574	99.5	99.6	KY322649
P7Ah3_1	*Acer pseudoplatanus*	*Cadophora orchidicola*	MT107569	99.1	99.6	KR230081
P8Ah1_1	*Acer pseudoplatanus*	*Cadophora orchidicola*	MT107570	99.5	99.6	AF486133
P9Ah4_7	*Acer pseudoplatanus*	*Cadophora orchidicola*	MT107578	99.3	99.6	KY322649
P17Es4_1	*Fraxinus excelsior*	*Cadophora orchidicola*	MT107609	99.5	99.5	KF646097
P18Es5_6-1	*Fraxinus excelsior*	*Cadophora orchidicola*	MT107616	99.3	100	AF486133
P18Es5_6-2	*Fraxinus excelsior*	*Cadophora orchidicola*	MT107623	99.3	100	AF486133
P2Es1_4	*Fraxinus excelsior*	*Cadophora orchidicola*	MT107608	98.8	98.8	KF646097
P3Es3_2	*Fraxinus excelsior*	*Cadophora orchidicola*	MT107621	98.7	98.7	KF646097
P6Es5_7	*Fraxinus excelsior*	*Cadophora orchidicola*	MT107602	98.5	99.3	KY271867
P10Ah4_2	*Acer pseudoplatanus*	*Cadophora* sp.	MT107615	97.5	98.8	JN859254
P11Ah5_6	*Acer pseudoplatanus*	*Cadophora* sp.	MT107622	97	98.7	JN859254
P17Ah5_1	*Acer pseudoplatanus*	*Cadophora* sp.	MT107588	97.3	98.6	JN859254
P9Ah3_6	*Acer pseudoplatanus*	*Cadophora* sp.	MT107601	97.5	98.8	JN859254
P2Es5_2	*Fraxinus excelsior*	*Cadophora* sp.	MT107614	97.5	100	JN859244
P16Ah4_2	*Acer pseudoplatanus*	*Cylindrocarpon* sp.	MT107581	99.4	100	KM248576
P13Ah2_2	*Acer pseudoplatanus*	*Ilyonectria destructans*	MT107604	99.8	100	MN540280
P18Ah5_2	*Acer pseudoplatanus*	*Ilyonectria destructans*	MT107611	100	100	MN540280
P2Ah4_1	*Acer pseudoplatanus*	*Ilyonectria destructans*	MT107624	100	100	MN540280
P3Ah1_2	*Acer pseudoplatanus*	*Ilyonectria destructans*	MT107617	99.8	100	MN540280
P14Es3_2	*Fraxinus excelsior*	*Ilyonectria destructans*	MT107625	99.8	99.8	MN540280
P7Es5_1	*Fraxinus excelsior*	*Ilyonectria destructans*	MT107594	100	100	MN540280

P12Fi3_2	*Picea abies*	*Ilyonectria destructans*	MT107595	99.8	99.8	MN540280
P5Fi3_1	*Picea abies*	*Ilyonectria destructans*	MT107580	99.8	99.8	MN540280
P8Fi3_1	*Picea abies*	*Ilyonectria destructans*	MT107589	100	100	MN540280
P8Es3_2	*Fraxinus excelsior*	*Ilyonectria* sp.	MT107603	100	100	MN385473
P3Fi2_2-1	*Picea abies*	*Mollisia* sp.	MT107629	99.2	99.8	JN225881
P11Ah1_2	*Acer pseudoplatanus*	*Neonectria* sp.	MT107590	100	100	KF428615
P12Ah5_1	*Acer pseudoplatanus*	*Neonectria* sp.	MT107596	99.3	99.6	KF428615
P14Es5_1	*Fraxinus excelsior*	*Neonectria* sp.	MT107618	100	100	KF428615
P18Es2_1	*Fraxinus excelsior*	*Neonectria* sp.	MT107610	99.8	99.8	KF428615
P8Ah1_2	*Acer pseudoplatanus*	*Pezicula ericae*	MT107587	99	100	KU516539
P8Es2_1	*Fraxinus excelsior*	*Pezicula ericae*	MT107627	98.5	99.2	KU516539
P16Ah1_1	*Acer pseudoplatanus*	*Pezicula melanigena*	MT107586	99.2	100	KU516542
P2Ah2_2	*Acer pseudoplatanus*	*Pezicula melanigena*	MT107612	98.8	100	LC206663
P17Es2_2	*Fraxinus excelsior*	*Pezicula melanigena*	MT107620	98.1	100	LC206663
P4Es5_1	*Fraxinus excelsior*	*Pezicula melanigena*	MT107584	98.5	99.4	NR_155611
P10Fi3_2b	*Picea abies*	*Pezicula melanigena*	MT107600	98.9	99.8	KU516542
P11Fi2_1	*Picea abies*	*Pezicula melanigena*	MT107579	98.9	99.8	KU516542
P12Fi5_1	*Picea abies*	*Pezicula melanigena*	MT107585	98.8	100	LC206663
P7Fi3_1	*Picea abies*	*Pezicula melanigena*	MT107598	99.2	100	KU516542
P10Es1_1	*Fraxinus excelsior*	*Pezicula radicicola*	MT107607	99.6	100	NR_155610
P14Es1_2	*Fraxinus excelsior*	*Pezicula radicicola*	MT107613	99.6	100	NR_155610
P2Es1_2	*Fraxinus excelsior*	*Pezicula radicicola*	MT107599	100	100	NR_155610
P3Fi5_2	*Picea abies*	*Pezicula radicicola*	MT107583	99.6	100	NR_155610
P6Es4_1	*Fraxinus excelsior*	*Phoma eupyrena*	MT107628	99.6	99.6	AJ890436
P2Ah3_6	*Acer pseudoplatanus*	*Phoma* sp.	MT107571	98.7	98.9	MF948641
P4Ah5_2	*Acer pseudoplatanus*	*Phoma* sp.	MT107572	98.8	99	MF948641
P5Ah3_1	*Acer pseudoplatanus*	*Phoma* sp.	MT107575	99.2	99.4	MF948641
P2Es4_1	*Fraxinus excelsior*	*Phoma* sp.	MT107565	98.7	98.9	MF948641
P2Es4_6	*Fraxinus excelsior*	*Phoma* sp.	MT107576	98.6	98.8	MF948641
P2Es4_7	*Fraxinus excelsior*	*Phoma* sp.	MT107577	98.8	99	MF948641
P4Es1_6	*Fraxinus excelsior*	*Phoma* sp.	MT107566	99.2	99.4	MF948641
P4Es4_1	*Fraxinus excelsior*	*Phoma* sp.	MT107562	99	99.2	MF948641
P4Es4_7	*Fraxinus excelsior*	*Phoma* sp.	MT107563	99	99.2	MF948641
P5Es2_6	*Fraxinus excelsior*	*Phoma* sp.	MT107564	99	99.2	MF948641
P3Fi1_1	*Picea abies*	*Phoma* sp.	MT107573	99	99.2	MF948641
P10Fi1_2	*Picea abies*	*Phomopsis phaseoli*	MT107630	96.2	98.3	EU272538
P10Fi2_2	*Picea abies*	*Phomopsis phaseoli*	MT107582	96	98.2	EU272538
P14Fi2_1	*Picea abies*	*Phomopsis phaseoli*	MT107592	96.1	98.4	EU272538
P2Fi1_2	*Picea abies*	*Phomopsis phaseoli*	MT107626	96.1	98.3	EU272538
P2Fi4_1	*Picea abies*	*Phomopsis phaseoli*	MT107619	96.1	98.4	EU272538

P3Fi5_1	*Picea abies*	*Phomopsis phaseoli*	MT107591	99.1	99.6	AF001018
P6Fi3_1	*Picea abies*	*Phomopsis phaseoli*	MT107605	96.2	98.3	EU272538
P6Fi4_1	*Picea abies*	*Phomopsis phaseoli*	MT107597	96.2	98.5	EU272538
P5Es5_6	*Fraxinus excelsior*	*Rhexocercosporidium* sp.	MT107567	98	99.2	MN124205
P5Es5_7	*Fraxinus excelsior*	*Rhexocercosporidium* sp.	MT107568	98.2	100	MN124203

[1] Species names according to Index Fungorum (http://www.indexfungorum.org/Names/Names.asp) (accessed on February 26, 2020).
